# Tools for self- or peer-assessment of interprofessional competencies of healthcare students: a scoping review

**DOI:** 10.3389/fmed.2024.1449715

**Published:** 2024-10-02

**Authors:** Sharon Brownie, Jia Rong Yap, Denise Blanchard, Issac Amankwaa, Amy Pearce, Kesava Kovanur Sampath, Ann-Rong Yan, Patrea Andersen, Patrick Broman

**Affiliations:** ^1^School of Health Sciences, Swinburne University of Technology, Hawthorn, VIC, Australia; ^2^School of Medicine and Dentistry, Griffith Health, Griffith University, Southport, QLD, Australia; ^3^Centre for Health and Social Practice and Centre for Sports Science and Human Performance, Waikato Institute of Technology, Hamilton, Waikato, New Zealand; ^4^College of Health, Medicine and Wellbeing, The University of Newcastle, Callaghan, NSW, Australia; ^5^Faculty of Health and Environmental Sciences, Auckland University of Technology, Auckland, Auckland, New Zealand; ^6^School of Health Sciences, Faculty of Health, University of Canberra, Canberra, ACT, Australia; ^7^Division of Health, University of Waikato, Hamilton, Waikato, New Zealand; ^8^School of Nursing, Midwifery and Paramedicine, University of the Sunshine Coast, Maroochydore, QLD, Australia

**Keywords:** interdisciplinary education, interdisciplinary communication, interprofessional collaboration, self-assessment, peer assessment, healthcare student

## Abstract

**Introduction:**

Healthcare professionals are expected to demonstrate competence in the effective management of chronic disease and long-term health and rehabilitation needs. Care provided by groups of collaborating professionals is currently well recognized as a more effective way to support people living with these conditions than routine, single-profession clinical encounters. Clinical learning contexts provide hands-on opportunities to develop the interprofessional competencies essential for health professional students in training; however, suitable assessment tools are needed to support student attainment of interprofessional competencies with self-assessment espoused as an important component of learning.

**Method:**

A structured approach was taken to locate and review existing tools used for the self-assessment and peer assessment of students’ competencies relevant to interprofessional practice.

**Results:**

A range of self- and/or peer assessment approaches are available, including formally structured tools and less structured processes inclusive of focus groups and reflection.

**Discussion:**

The identified tools will usefully inform discussion regarding interprofessional competency self- and peer assessment options by healthcare students participating in a broad range of clinical learning contexts.

**Conclusion:**

Self- and/or peer assessment is a useful approach for those seeking to effectively enhance interprofessional learning and measure the attainment of related competencies.

## 1 Introduction

An increasing focus on interprofessional education is needed as student health professionals prepare for a context of increasing health complexity, non-communicable disease, co-morbid conditions, and aging populations (1, 2). Programs of care provided by professionals working together are currently well recognized as a more effective way to support people living with these conditions than routine, single-profession clinical encounters. Patients increasingly expect a broader and more coordinated approach to their care (2, 3). Recognition of the need for team-based collaborative care and interprofessional education is not new as shown by documents such as the *World Health Organization (2010) Framework for Action on Interprofessional Education and Collaborative Practice* (4). Subsequently, curriculum content focused on the development of interprofessional competencies is an increasingly expected component of health professional education (5, 6). Interprofessional competencies form the basis of safety, quality, and patient-centeredness in team collaboration contexts (7, 8). These competencies include team participation, leadership, and communication (9). Interprofessional competence also includes soft skills such as attitudes, values, ethics, and teamwork, facilitating difficult conversations, multi-party communication, and trust building (8, 10, 11). Interprofessional competence is required for effective modern healthcare practice but all too often, various barriers get in the way of teaching interprofessional (IP) competencies, given that training usually involves professionals working in isolation using their own discipline knowledge base (1, 12).

Clinical learning environments, or contexts in which health professional programs are taught and practice placements occur, provide hands-on opportunities to support student attainment of IP competencies. Best practices in clinical education involve continuous feedback as a critical link between teaching and assessment and essential in supporting the educational process (13, 14) with self- and peer assessment espoused and regularly used as an important component of the learning sequence (15, 16). Twenty years ago, Ward et al. (2002) reported that reflection on practice using self- and peer assessment is not without difficulties, raising concerns such as issues in objectivity and reliability of students assessing their own performance (17), and debates have persisted since that time (18). Despite these concerns, self-assessment is widely implemented as an educational learning process (16). In the face of concerns, suitably validated self- and peer assessment tools are needed to guide best practices, complement faculty assessment processes, and effectively maximize learning (19).

Previous reviews identifying interprofessional assessment tools for use with prelicensure students have focused on post-placement or post-intervention assessment of IP competency (20) or the identification of tools for use by faculty in the assessment of student IP development (21). This inquiry aimed to locate assessment tools and assessment processes used by prelicensure healthcare students for the self- and peer assessment of IP competency attainment in clinical learning contexts, including the potential for use within an interprofessional student-led clinic. Student-led clinics (SLCs) are a unique option for the provision of practice placements in health professional programs (22). They are used with increasing frequency to enhance the opportunity and experience for prelicensure students in hands-on practice, especially in primary healthcare settings, while also providing benefits to service users and communities (22–24). SLCs May involve students from single professions (22) or May be interprofessional in nature (25, 26). Within both general clinical learning contexts and SLCs, tools May be used to assess either individuals or whole teams in interprofessional competencies.

This study sought to understand what assessment tools and self/peer assessment processes have been used by prelicensure healthcare students during interprofessional self-assessment and peer assessment processes in clinical learning environments with two or more health professionals working together. In developing this search, we noted that “tools,” “techniques,” instruments,” and “scales” are frequent terms used interchangeably in the literature (27–29). Definitions are closely aligned and often contradictory (30, 31). For this review, the term ‘tool’ is reported for consistency. Consistent with our research question, we also report processes that did not include the utilization of formally developed ‘tools’ but also other means such as self- or peer reflection and focus group discussions to measure, assess, or reflect on interprofessional competency development.

The inquiry focused on student self- and peer assessment versus assessment undertaken by teaching faculty and on the self-assessment of interprofessional competencies versus profession-specific competencies. The review aimed to answer the following questions:

- What tools and self−/peer assessment processes have been used by prelicensure healthcare students to undertake self- and/or peer assessment of interprofessional competencies in an interprofessional clinical learning context (contexts in which health professional programs are taught and practice placements occur) with two or more health professions working together?

## 2 Method

### 2.1 Reporting guideline

A scoping review was considered most appropriate for investigating the research question as this topic has not yet been comprehensively reviewed. In such instances, scoping reviews are suitable to provide a general overview of available evidence (assessment tools) as a precursor to more detailed inquiry (32). A scholarly approach was undertaken in conducting the review using the Preferred Reporting Items for Systematic Reviews and Meta-Analyses Extension for Scoping Reviews (PRISMA-ScR) statement (33).

### 2.2 Eligibility criteria

This review sought primary studies using qualitative, quantitative, or mixed methods for assessing interprofessional competencies. Specifically, we searched for studies involving healthcare students (from two or more professions working together) at any level of study, participating in interprofessional education activities, and utilizing tools to self-assess interprofessional competence or peer assess other students. The search focused on prelicensure students. Publications in which participants included registered health professionals and those with initiatives to maintain registration or undertake continuing professional development were excluded. Studies that assessed IPE programs more broadly and in which tools were used for the primary purpose of program evaluation, as opposed to specifically assessing student IPE competencies as a result of such programs, were excluded. The selection criteria are summarized in Table 1.

**Table 1:** Inclusion and exclusion criteria.

Inclusion criteria	Exclusion criteria
Self-assessment Peer assessment Healthcare students at any level of study Interprofessional education involving two or more professions working together Assessment of interprofessional competence	Registered health professionals Initiatives to maintain registration Continuing professional development Assessment of IPE programs Studies involving one profession only Studies using self or peer assessment for program evaluation only

### 2.3 Information sources

The literature search was completed in May 2023 and updated in November 2023 followed by analysis and write-up. Four electronic databases, ProQuest, ERIC, Medline, and Embase, were searched for literature published in the 25 years preceding the search date. By focusing on the last 25 years, the review aligns with the transfer in various nations of hospital-based education to university-based education and captures the most relevant and impactful developments in the field of Interprofessional Education and Collaborative Practice (IPECP). This approach allowed us to concentrate on the period during which these concepts gained significant traction, thereby providing a more focused and pertinent analysis.

### 2.4 Search process

The search strategy was guided by the research question and the inclusion/exclusion criteria, focusing on three broad concepts: healthcare student, peer- and self-assessment, and interprofessional competence, with refinement through MeSH headings in Medline. The initial search in ERIC used the following keywords: [(Pre-registration OR Pre-licensure) AND (Healthcare student OR Healthcare student) AND (postgraduate OR undergraduate) AND (Evaluate OR Assessment OR assessing OR assess OR outcome OR outcomes OR examin* OR evaluate) OR (measurement OR measure OR measuring) AND (Competenc* OR Competent) AND (interprofession*) AND tools]. The search strategy was then tailored to each database accordingly. Google Scholar was specifically used to search for gray and narrative literature that might have been missed in the focused search as well as to explore reference lists of relevant primary papers in the database search.

### 2.5 Study selection

Search results were imported into Covidence® (34), an online software for review data management and screening, which automatically removed duplicates. Initial screening of the titles and abstracts was conducted by two sets of independent reviewers. Disagreements regarding paper inclusion were resolved by discussion between a third and fourth reviewer.

Full texts of included studies were then reviewed by two sets of independent reviewers. Discrepancies and conflicts were resolved by a third reviewer.

### 2.6 Data extraction

Two reviewers independently extracted relevant data from the included studies via Covidence for review and discussion by all authors. This information encompassed the following parts: the characteristics of studies (publication year, country, study design, sample population, and size), participant features (student professional field and level of study), and characteristics relating to the intervention, control, and outcome measures (IPE, interprofessional competency, and self/peer assessment tools). Any conflicts that arose between the reviewers were resolved by consensus.

Focused effort with significant rereading and team discussion was needed to locate studies directly relevant to the research questions. This was because significant literature was identified where students undertook self-assessment activities using published scales; however, on close examination, the student self-assessment data were used to inform tutor evaluation of the effectiveness of the IPE program or intervention rather than for the students’ personal assessment, discussion, and reflection. Examples include (35–37). Articles that used student self-assessment data purely to inform program evaluations were excluded in the review process because this review directly related to the question ‘what tools and self/peer assessment processes have been used by prelicensure healthcare students to undertake self- and/or peer assessment of interprofessional competencies in an interprofessional clinical learning context with two or more health professions working together?’ Some studies had a dual-purpose use of the student self-evaluation data—to inform both student self-evaluation and program evaluation. If data were available for student self-assessment and/or reflection, the study was identified as relevant to this review.

## 3 Results

**Figure 1:**
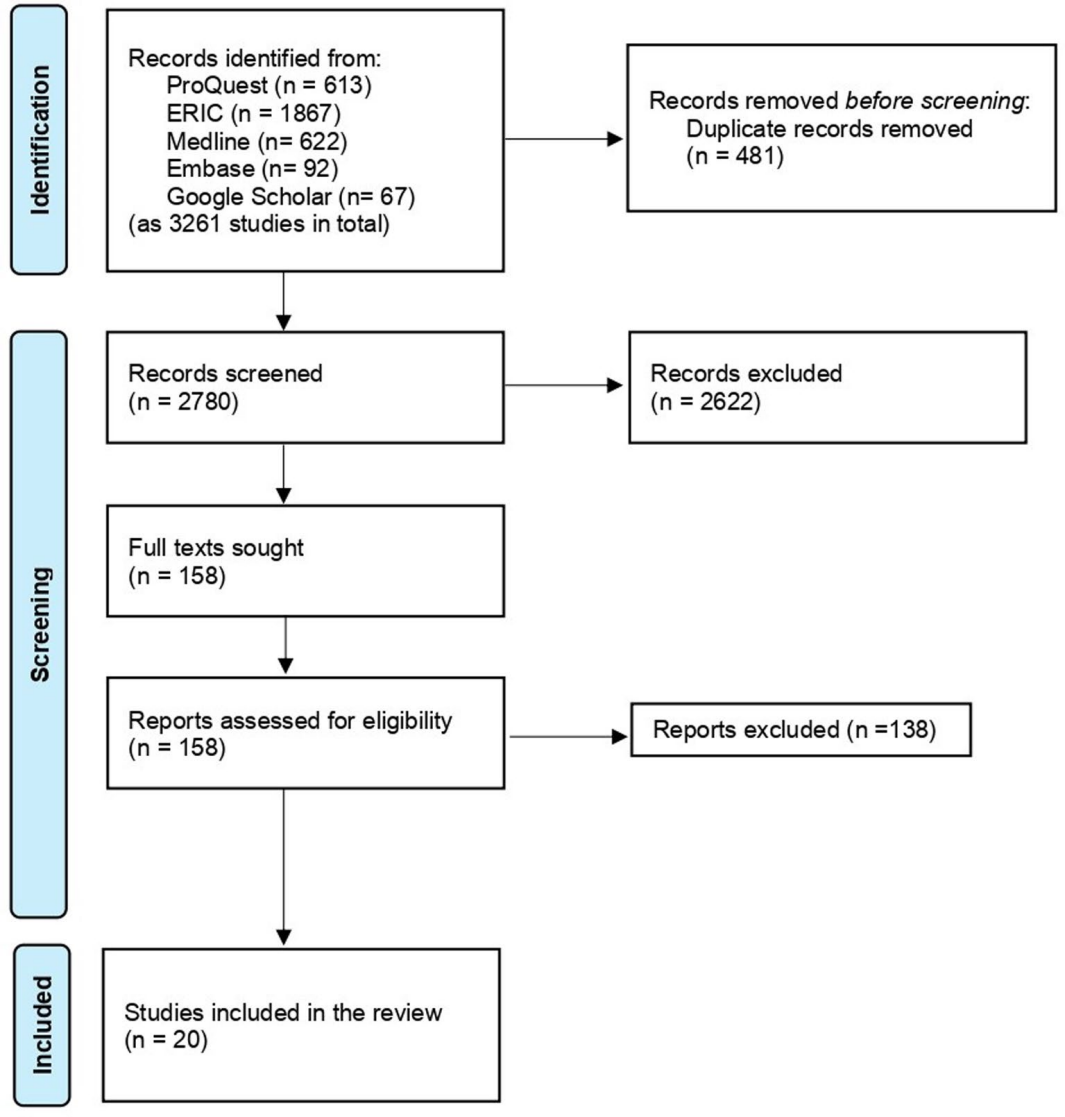
Literature search and PRISMA diagram.

Twenty studies were identified of direct relevance to the review question (see Figure 1).

### 3.1 Characteristics of included studies

Table 2 provides a summary of the characteristics of each study selected for inclusion in this review. Studies were identified across a 25-year timeframe from 2009 and involved quantitative, qualitative, and mixed-methods approaches. A wide range of health professions were reported in the selected studies with nursing and pharmacy the most frequently noted. Studies originated from the United States, Canada, Australia, and the United Kingdom, with the highest number (14 or 70%) having been published in the United States.

**Table 2:** Characteristics of included studies.

Category	Number of papers	Percentage^1^
Assessment design		
Qualitative	2	10%
Quantitative	9	45%
Mixed	9	45%
Self or peer assessment		
Self-assessment	16	80%
Both peer and self-assessment	4	20%
Student professions included		
Nursing (registered or nurse practitioner)	14	70%
Medicine	8	40%
Pharmacy	11	55%
Social Work	6	30%
Physical Therapy/Physiotherapy	9	45%
Dental	2	10%
Occupational Therapy	6	30%
Physician Assistant	4	20%
Speech and Language Therapy/Pathology	5	25%
Public Health	3	15%
Audiology	2	10%
Other professions (1 study each)^2^	11	55%
Year published		
2000–2009	2	10%
2010–2019	10	50%
2020+	8	40%
Country of publication		
USA	14	70%
Canada	3	15%
Australia	2	10%
UK	1	5%

^1Percentage of student professions sums to greater than 100 as studies included students from two or more professions. 2Clinical Psychology, Radiography, Cardiac Physiology, Dietetics, Nutrition, Anaesthesiologist Assistant, Human Services, Recreational Administration, Graduate Counseling, Exercise Science, Human Kinesthetics.^

### 3.2 Analysis of included studies

For the purposes of this review, assessment tools identified May have been used for either self- or peer assessment, with results having been provided to students for the purposes of learning assessment, rather than being used by educators or researchers for program or course evaluation. Table 3 lists each study and provides information about the number of participants, the intervention (IPE learning activity), the participating student population, and the specific assessment tools used. Note that where assessment was undertaken by *instructors* or *faculty* in conjunction with self-assessment or peer assessment in a given study, these tools are not listed. For example, Begley et al. (2019) also used the Creighton Interprofessional Collaborative Evaluation (C-ICE) instrument, a “25-point dichotomous tool in which the evaluator awards one point if the interprofessional team demonstrates competency in a 2 specific area, or no point for failure to do so” (p. 477). Because this tool involves evaluator 3 (not self- or peer-) assessment, this tool is not listed or considered further here (38).

**Table 3:** Interprofessional education self/peer assessment tools.

Study	Participants	IPE activity and population description	Assessment design	Outcome/s assessed	Self-assessment IPE tool/s	Peer assessment IPE tool/s
Anderson, 2006	126	Interprofessional clinical teaching workshop of patient during an acute hospital episode Pre-registration (above first-year) clinical psychology, occupational therapy; medical; nursing; physiotherapy; pharmacy, radiography, cardiac physiology; dietetics; and speech and language students	Mixed	Interprofessional competencies in knowledge, skills and attitudes of team working	(Pre) 5-point Likert scale questions recording student hopes, concerns, and expectations on the learning event (Post) 19 5-point Likert scale questions, on the structure, organization of the session, and the teaching methodology + open-ended comments on the best and worst aspects	N/A
Dobson, 2009	134	Interprofessional quality improvement (QI) activity Undergraduate University of Saskatchewan nursing (years 2 and 4), nutrition (year 2); pharmacy (year 3), and physical therapy (year 3) students	Quantitative	Interprofessional team knowledge, attitudes, and beliefs	16 7-point Likert scale questions on interprofessional self-reflection	(Post) Group evaluation score, including 9 7-point Likert scale questions anchored by diametrically opposing statements about the functioning of their team + Open-ended comments
Dubouloz, 2010	1	Interprofessional Rehabilitation University Clinic in Primary Health Care	Mixed	Attitudes toward interprofessional learning and collaboration	RIPLS: readiness for interprofessional practice DMILTS: description of a meaningful Interprofessional learning situation tool	N/A
Guitard, 2010	15	Interprofessional Rehabilitation University Clinic in Primary Health Care Prelicensure audiology; occupational therapy; physiotherapy and speech-language pathology students	Qualitative	Level of knowledge and perceptions about the importance of interactional determinants of collaboration	Written, semi-structured questionnaire (post-)	N/A
Seif, 2014	332	Interprofessional service-learning course and student-run free clinic (SRFC) Pre-clinical physical therapy; occupational therapy; physician assistant; medical; pharmacy students	Quantitative	Interprofessional perceptions and attitudes and perceptions of clinical reasoning skill	IEPS: interdisciplinary education practice learning scale RIPLS: readiness for interprofessional practice	N/A
Sevin, 2016	15	Collaborative Competencies in Service Learning Course Undergraduate nursing and social work and graduate professional pharmacy students	Quantitative	Interprofessional education collaborative competencies	IPEC (42-item) Interprofessional education collaborative competency self assessment tool NB: an updated version 3 of the IPEC competencies was released on November 2023	N/A
Simko, 2017	60	Interprofessional Pain Education Course Senior nursing and year 5 pharmacy students who attended an elective course	Quantitative	Perspectives on interprofessional teamwork and collaboration	IEPS: interdisciplinary education perception scale CSACD: collaboration and satisfaction about care decisions	N/A
Nash, 2018	191	A multifaceted educational program consisting of technology-enhanced delivery as well as interactive exercises in a joint health assessment course University of Louisville nurse practitioner (year 2 of a 2-year program) and dental (year 1) students	Quantitative	Knowledge of Interprofessional education core competencies; attitudes toward interprofessional education; attitudes toward teamwork; self-efficacy in functioning as a member of an interdisciplinary team	A 17-item measure of student understanding of IPE core competencies (based on Interprofessional Education Core Competencies); RIPLS: Readiness for Interprofessional Practice T-TAQ (24 of 30 items only) Team STEPPS Teamwork Attitudes Questionnaire SEF-MIT: Self-Efficacy in Functioning as a Member of an Interdisciplinary Team Scale	N/A
Seaman, 2018	62	Interprofessional clinical placement in ambulatory care Final year students enrolled in Master of Nursing Science or MBBS (medicine)	Mixed	Interprofessional socialization	ISVS: Interprofessional Socialization and Valuing Scale Open-ended questionnaires: (Pre) anticipated learning (Post) student perspectives on the impact of experience	N/A
Begley, 2019	162	PE telehealth cases Creighton University pharmacy (years 1–3) and physician assistant (year 2) students	Mixed	Interprofessional student team performance	TSS: Team Skills Scale Written reflections (pharmacy students)	N/A
Leithead, 2019	152	High-fidelity simulation (HFS) operating room (OR) interprofessional team training Senior medical, undergraduate nursing, and nurse anesthesia students	Quantitative	Attitudes toward interprofessional learning and collaboration	RIPLS: Readiness for Interprofessional Practice 15 6-point Likert scale questions on interprofessional teamwork	TAS
Roberts, 2019	45 students and 51 health professionals	Study 1: IPE workshop on pediatric head injury; Study 2: IPE workshop on error disclosure Recreational admin; nursing; social work; speech pathology; pharmacy and public health graduate and undergraduate students; and health professionals	Quantitative	Interprofessional competencies	IPEC (42-item) interprofessional education collaborative competency self-assessment tool	N/A
August, 2020	39	Community Homeless Interprofessional Program (CHIP) or Diabetes Education Wellness (DEW) Wayne State University health students of pharmacy (years 1–3), medical (years 1–2), social work (various years), physical therapy (years 1–3)	Quantitative	Interprofessional socialization	ISVS: Interprofessional Socialization and Valuing Scale	N/A
Johnson, 2020	68	An experiential interprofessional education program based on the ICF model	Qualitative	Interprofessional education collaborative competencies	Open-ended pre- and post-survey	N/A
		Graduate professional physical therapy; physician assistant; pharmacy students				
Pawlowska , 2020	111	Baby Day: a pediatric IPE activity Graduate physical therapy, occupational therapy and speech language therapy; and undergraduate nursing students	Mixed	Interprofessional collaborative competencies	5-point Likert scale questions on the extent activity allowed students to meet interprofessional learning goals of activity Guided reflective writing assignment	N/A
Porter, 2020	11	Effects of Experiential Competency-Based Interprofessional undergraduate course Pre-professional human services; public health and nursing students	Mixed	Interprofessional competencies	Modified IPEC (‘I’ replaced with ‘the team’) + Open-ended prompts describing reflections pertaining to team experiences	N/A
Nieuwoudt, 2021	77	Interprofessional simulation sessions, representing a GEM ward Pre-registration (year 1) nursing and occupational therapy students	Mixed	Interprofessional practice competencies	IPEC (16-item); interprofessional education collaborative competency self assessment tool Focus groups	N/A
Timm, 2021	26	An interprofessional faculty + student-led clinic Undergraduate and graduate nursing; social work; exercise science; graduate counselor education students	Mixed	Interprofessional practice competencies	IPEC (42-item); interprofessional education collaborative competency self assessment tool ISVS: interprofessional socialization and valuing scale Focus group interviews	N/A
Vyas, 2021	1,099	A telehealth-based interprofessional education (IPE) experience Teams of one doctor of osteopathic medicine and one or two doctor of pharmacy students	Mixed	Interprofessional collaborative competencies	ICCAS: Interprofessional Collaborative Competencies Attainment Scale	A peer evaluation on the TEAMMATES app V7.8.0, providing feedback to their team member(s)
Earnest, 2022	1,357	Classroom-based IPE course Anaesthesiologist assistant, dental medicine, medical, nursing, pharmacy, physical therapy, physician assistant; public health and social work students	Quantitative	Team member effectiveness and collaborative competency	CATME: Comprehensive Assessment of Team Member Effectiveness	CATME

The 12 specific tools in Table 3 have been used for self-and/or peer assessment of interprofessional competencies across the twenty included studies are shown in Table 4.

**Table 4:** Tools used for self or peer assessment of interprofessional competencies.

Scale	Full name	Description	Studies used	Validated?
ISVS	Interprofessional socialization and valuing scale	A 24-item, 6-point Likert scale measuring beliefs, behaviors, and attitudes underlying interprofessional socialization (assumptions and worldviews, knowledge and skills concerning collaborative teamwork, values, and identities)	Seaman, 2018 August 2020 Timm, 2021	Yes
IEPS	Interdisciplinary education perception scale	A 12−/18-item, 6-point Likert scale measuring perceptions and attitudes about competency and autonomy, the need for cooperation, and the perception of actual cooperation	Seif, 2014 Simko, 2017	Yes
RIPLS	Readiness for interprofessional learning scale	A 19-item, 5-point Likert scale measuring perceptions of knowledge, skills, and attitudes regarding readiness to learn with other healthcare professionals	Dubouloz, 2010 Seif, 2014 Nash, 2018 Leithead, 2019	Yes
IPEC	Interprofessional education collaborative competency self-assessment tool	A 32- or 16-item (revised), 5-point Likert scale measuring competencies related to collaborative practice based on core competency statements developed by the Interprofessional Education Collaborative (IPEC, 2011)	Sevin, 2016 Roberts, 2019 Timm, 2021 Nieuwoudt, 2021	Yes
ICCAS	Interprofessional collaborative competencies attainment survey	A 20-item, 5-point Likert scale measuring perceived skills in communication, collaboration, roles and responsibilities, collaborative patient-family-centered approach, conflict management/ resolution, and team functioning. Completed once after IPE training, rating abilities two times: once as recalled prior to training, and again now that training is done	Vyas, 2021	Yes
TSS	Team skills scale	A 17-item, 5-point Likert scale measuring self-assessment of skills required to work effectively on an interprofessional care team (interpersonal skills, discipline-specific skills, and geriatric care skills)	Begley, 2019	Unclear
CATME	The comprehensive assessment of team member effectiveness	A 5-item, 5-point Likert scale measuring team member contributions in five areas based on team effectiveness literature (contributing to teamwork, interacting with teammates, keeping the team on track, expecting quality, having relevant knowledge/skills/abilities)	Earnest, 2022	Yes
CSACD	Collaboration and satisfaction about care decisions	A 9-item, 7-point Likert scale was used to assess the quality of interaction in making care decisions and satisfaction with the decision-making process in the health setting (7 related to collaboration)	Simko, 2017	?
DMILST	Description of a meaningful interprofessional learning situation tool	Short-answer, open-ended questions to identify: (1) knowledge of other professions gained, (2) learning experiences about four key determinants of collaboration, and (3) students’ perceived impact of the interprofessional application on client care, their learning, and educator–clinicians’ supervision	Dubouloz, 2010	No
T-TAQ	Team STEPPS teamwork attitudes questionnaire	A 30-item, 5-point Likert scale measuring five core components of teamwork: team structure, leadership, situation monitoring, mutual support, and communication	Nash, 2018	Yes
SEF-MIT	Self-Efficacy in functioning as a member of an interdisciplinary team scale	A 17-item, 4-point Likert scale measuring self-efficacy in two core competency statements developed by the Interprofessional Education Collaborative (IPEC, 2011)—roles/responsibilities and interprofessional communication	Nash, 2018	No
TAS	Teamwork assessment scale	A 14-item, 5-point Likert scale observational tool measuring overall team functioning, based on a theoretical model of teamwork	Leithead, 2019	Yes

The origins of the frequently used tool can be found in USA which has a strong history of formally established interprofessional learning collaboratives. For example, the ISVS Scale was developed by the Minnesota-based National Centre for Interprofessional Education and Practice (39), the IPEC scale was developed by the Washington-DC-based Interprofessional Education Collaborative. It appears that US educators have the autonomy to choose and utilize various tools or to construct their own approaches. The UK hosts CAIPE—the Centre for the Advancement of Interprofessional Practice and Education established in 1987 to drive interprofessional practice in health (40). However, UK educational providers appear to have less autonomy as UK-based regulators mandate the actual competencies, which must be addressed by each profession. We can only speculate that this May be why only one UK-based manuscript appeared in this search.

The 12 assessment tools vary in different ways, although 11 of the 12 tools are quantitative, Likert-scale measures, with the exception being the Description of a Meaningful Interprofessional Learning Situation Tool developed by Dubouloz et al. (2010) to capture students’ perspectives qualitatively, via open-ended questions. This is the only specific qualitative tool used (41); however, other studies also adopted less structured approaches to self and peer assessment, such as the use of focus groups or written reflection tasks. Table 4 only includes the 14 studies in which formal tools were utilized. Utilization of tools was most frequently reported in mixed-methods studies, in conjunction with a more structured, quantitative approach utilizing a scaled tool (38, 42–44). Two studies adopted a solely qualitative approach, with students undertaking self-assessment via reflective written questionnaire/open-ended survey (post-test-only and pre-and-post, respectively) (45, 46).

Among quantitative approaches, the most frequently used tool was the IPEC (43, 44, 47, 48). This is a 5-point Likert scale tool based on the well-known core competency statements developed by the Interprofessional Education Collaborative (IPEC, 2011), a U.S. collaboration involving peak bodies from six health disciplines. An early 42-item scale includes 8 to 11 items for each of the four key domains in the statement (values and ethics, roles, and responsibilities, interprofessional communication, and teams and teamwork), although Nieuwoudt et al. (2021) used a shortened 16-item scale and Porter et al. (2020) modified the scale to use ‘the team’, instead of ‘I’ for each of the competencies (43, 49). Note that an updated version 3 of the IPEC competency standards was released in late 2023 shortly after the conclusion of the search process associated with this manuscript (50). The updated version is available as a resource to inform future studies.

Three of the included studies used ISVS for students to self-assess attitudes, values, and beliefs about the value of interprofessional socialization (44, 51, 52). The second most used tool was the Readiness for Interprofessional Learning Scale (RIPLS), used by Ref. (41, 53–55). However, it is important to note that in each of these cases, this tool was used alongside one or more other tools for assessing competencies. In each study including RIPLS the decision to include it is not explained. While a valid and reliable tool, RIPLS (56), was not designed to be an outcome or impact measure. It is designed to measure attitudes toward IPE *before* starting an IPE intervention. However, it is appropriate to include studies, which have utilized the RIPLS scale on the basis that this scale measures attitudes and values as regards interprofessional educational activities. Collaborative attitudes and values, including the attitude and openness to follow leaders within a team, are important interprofessional competencies (10, 11, 57), and students’ awareness of their own situation is an important part of interprofessional learning. In considering the decision to include studies using the RPILS scale in the findings of this search, it is important to reflect on the variance among the 12 tools highlighted in Table 4. Assessment is a multivariate process. One size does not fit all. Thus, a selection of different types of tools and processes for differing settings is both valid and useful.

Considering differences is also important to differentiate between the most common type of tool, which measures individual competencies (whether for oneself or one’s peers), and those which measure competencies overall, for a team. Most located studies used individual and personal scales, but there were some examples of scales or tools which measured overall team functioning, skills, or approaches. These include the CATME used in Ref. (58) originated in engineering, and which involves individuals assessing self- and team-member contributions to a team, and the Teamwork Assessment Scale used in Ref. (53), which assesses team functioning in a given situation (items include ‘the team roles were distinct without ambiguity’, for example). The CASCD scale used by Ref. (59) measures perceptions of team interaction and satisfaction with decision-making and is thus also more situational and team functioning focused than a scale of individual skills, knowledge, or experience.

Other studies used (either solely or alongside named tools) in-house constructed Likert-scale instruments not listed in Table 3 (51, 57, 60). Validation for these tools, particularly a detailed description of their psychometric properties, was typically lacking (61). Likert-scale ranked approaches were typically used in a pre- and post-design before and after the intervention, but there are also examples of retrospective, post-then-predesign where participants recalled prior knowledge after the fact (48) and ICCAS used by Vyas et al. (2021) is designed to be completed only once, rating abilities after training and also as recalled previously (62). Overall, there was significant variability in the approaches to self- and peer- assessment undertaken by students in these contexts and in the tools and processes used.

## 4 Discussion

Effective assessment should be designed in a multifaceted manner and include a variety of formative and summative assessment activities and continuous learner feedback with each assessment activity designed to build, test, and affirm learner capability and expand understanding. Using more than one assessment type helps give students a range of ways to demonstrate what they have learned and what still needs to be learned (63). Among the wide milieu to be learned by student health professionals, interprofessional insight and practice capabilities are increasingly important as populations age and levels of chronic and complex care priorities increase (2).

A recent review reported results of a search designed as a resource of interprofessional assessment tools used by faculty (21). This search was designed to complement this study by locating and providing a pointer to tools and processes available for student self-assessment and peer assessment of interprofessional understanding and capability. As highlighted, the search identified studies utilizing and reporting formally developed IP self- and peer assessment tools along with other studies reporting processes such as focus groups and reflection—the benefit being an identification of a broad range of resources that can used to engage with students and their peers and enhance IP related learning among health professional students during their learning experiences. A significant benefit of self- and peer assessment is the extent to which these processes increase student understanding of their current capabilities and learning needs (58, 64).

### 4.1 Self- and peer assessment tools

Despite concerns in the literature about the objectivity and reliability of students assessing their own performance and/or that of their classmates (17, 18), peer and self-assessments have been shown to significantly contribute to the expansion of student capability and positive learning outcomes (64–66). The argument that self-assessment May be unreliable, inflated, and/or biased can be mitigated by including others (for example, peers, colleagues, and clients) in the assessment of self (67). Thus, the review has searched for examples using both self- and peer assessment tools and processes with both noted as being complementary to each other (65).

Documented benefits of self-assessment include the growth that occurs when students learn how to assess their own competencies and/or those of their peers. This includes increased ‘deep-level’ learning, critical thinking, and problem-solving skills (66). Reported benefits also include growth in self-awareness and the transition from tutor-directed learning, to self-directed learning, and ultimately, autonomous, reflective practice (66).

Timing of assessment and the benefits of repeating assessments are important considerations. Students May rate themselves inappropriately high before their learning experience and score lower in terms of comfort or ability after the placement, once they have greater insight into their capabilities and have been provided with an opportunity to reflect (51, 68). Self-assessment has also been reported as more likely to be inflated among first-year students with further instruction and reflection recommended to moderate over-confidence and self-bias among novice learners (69).

Aside from the use of formalized tools to facilitate self and/or peer assessment, verbal or written reflection and engagement in focus groups provide the opportunity for students to safely contemplate and recognize their own strengths and weaknesses and, as such, is a valuable aid to learning. Benefits also include reported increases in empathy, comfort in dealing with complexity, and engagement in the learning process (45, 46, 70, 71).

### 4.2 Assessment as a comprehensive concept

*“Effective assessment is more like a scrapbook of mementos and pictures rather than a single snapshot.”*

Wiggins and McTighe, 2005, p 152

**Figure 2:**
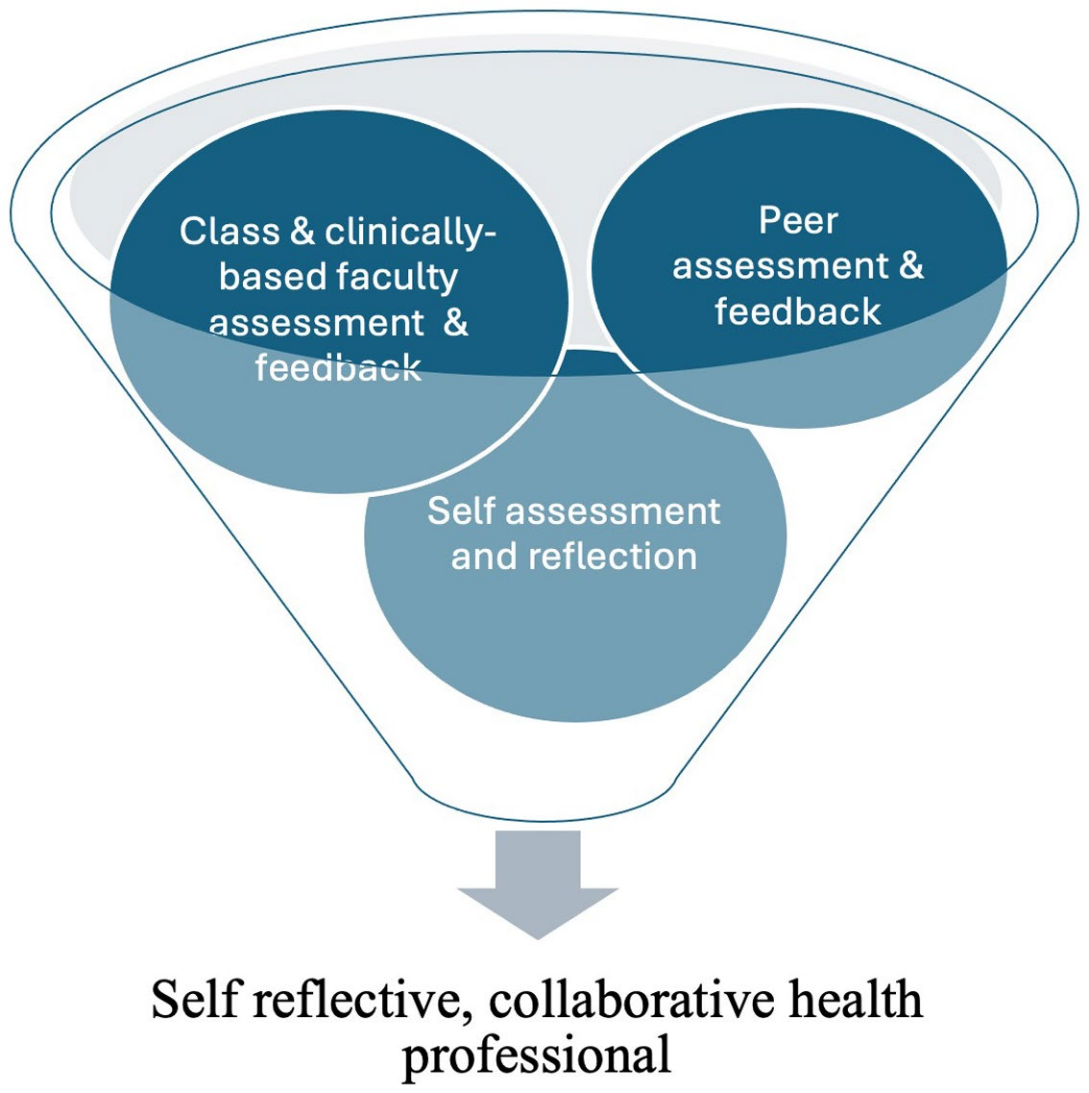
A comprehensive approach to assessment.

Multiple methods are needed to best capture the major aspects of knowledge and competency acquisition among student health professionals (72, 73). While the search has successfully identified self and/or peer assessment options for educators and their learners, it is important to position these within a broader suite of assessment options to maximize the development of a self-reflective health professional. Figure 2 illustrates the comprehensive nature of student assessment and the multivariate approach outlined by Wiggins and McTighe (2005), which is needed to support the development of critically thinking, self-reflective practitioners (63).

Blue et al. (2015) have noted that the lack of progress relating to the assessment of interprofessional competencies continues to create challenges for educators. Various studies conducting assessments have focused on learner attitudes toward IPE as opposed to learner IP knowledge or skill (61, 74, 75). Moreover, existing tools lack sufficient theoretical and psychometric development (61, 75). The Readiness for Interprofessional Learning Scale (56) and the Interdisciplinary Education Perception Scale (IEPS) (76) have been widely used, for both faculty (21) and student self and/or peer assessment (55), and other tools or scales have been locally developed to meet specific institutional goals and objectives (43, 49, 74). Blue et al. (2015) and Nieuwoudt et al. (2021) found that few programs reported systematic processes for evaluating individual student’s skills and behaviors related to interprofessional collaboration. It is clear that rigorous assessment and evaluation methods, standardized and widely used tools, and longitudinal assessment from diverse contexts are needed if the field of IPE is to advance and align with the demands of changing clinical care systems.

### 4.3 Need for further research

More study is needed to investigate the strengths and merits of qualitative scales versus more qualitative approaches in the assessment of interprofessional competency and within the suite of currently available self and/or peer assessment options. Reflective, deep dive approaches—both have been used in the literature, but little seems to have been done to reconcile them, test the value or otherwise of one over another or within mixed approaches (74) What is clear is that for the field to progress, there needs to be some consensus agreement on which measures to use to most effectively support learning.

## 5 Limitations

This study focuses on studies reporting self-assessment and peer assessment processes. Findings identify considerable variance among the identified tools and processes and the ways in which they were utilized. Several studies undertook a case study approach or included small cohorts only, so results May not be comprehensive or generalizable. As a detailed description of learning outcomes and psychometric properties of the results was typically lacking, it is not possible to make evidence-based comparative comments about which of the individual tools and/or processes as the most effective aids for learning. Thus, readers are encouraged to consider the recommendations in conjunction with the combination of assessment and feedback processes available to assess interprofessional readiness, capability, and competence and aid student learning.

## 6 Conclusion/recommendations

This review has identified a range of self- and peer assessment tools and processes to usefully contribute to the assessment of interprofessional competencies. Findings highlight the option of using a range of self and/or peer assessment approaches including formally structured tools and less structured processes, inclusive of focus groups and reflection. Discussion recommends that results identified within this search be used to complement tools, which can be used by faculty and others within a broader mosaic of assessments designed to support learning and the development of competent, self-reflective beginner practitioners. As such, the research provides a useful resource for seeking to effectively enhance interprofessional learning and competencies attainment. Of note is the conclusion that there is still more study to be undertaken in this area including the need for greater clarity and consensus agreement about definitions, tools, and the most appropriate measurement approach.

## Data Availability

The raw data supporting the conclusions of this article will be made available by the authors, without undue reservation.
